# Psychometric properties of patient-reported outcome measures to assess premenstrual syndrome/premenstrual dysphoric disorder in japanese: a systematic review using the COSMIN methodology

**DOI:** 10.1186/s41687-025-00910-4

**Published:** 2025-06-20

**Authors:** Kaori Tsuyuki, Miho Egawa, Takuma Ohsuga, Yumie Ikeda, Yoshitake Takebayashi, Hideki Sato, Masaki Mandai

**Affiliations:** 1 Department of Gynecology and Obstetrics, Kyoto University Graduate School of Medicine, Kyoto, Japan; 2 UMISORA Clinic Department of Gynecology, Kyoto, Japan; 3 Department of Health Risk Communication, Fukushima Medical University School of Medicine, Fukushima, Japan; 4 Department of Disaster Psychiatry, Fukushima Medical University School of Medicine, Fukushima, Japan

**Keywords:** Premenstrual syndrome, Premenstrual dysphoric disorder, Patient-reported outcome measures, Measurement properties, COSMIN, Systematic review

## Abstract

**Background:**

Patient-reported outcome measures (PROMs) are important for assessing premenstrual syndrome (PMS) and premenstrual dysphoric disorder (PMDD) to effectively capture subjective symptom burden and evaluate treatment effectiveness in clinical and research settings. This systematic review evaluated the psychometric properties of PROMs used to assess PMS/PMDD in Japan.

**Methodology:**

A systematic literature search was conducted in the MEDLINE, CINAHL, Cochrane Library, and Ichushi-Web databases. The COnsensus-based Standards for the selection of health Measurement Instruments (COSMIN) methodology was used to assess the methodological quality and measurement properties of the included PROMs.

**Results:**

A total of 13 studies that evaluated 12 versions of 11 unique PROMs were included. PROMs were categorized as recall-based (*n* = 9, 69%) or daily recording scales (*n* = 4, 31%). The structural validity and internal consistency were relatively well evaluated for most scales. However, evidence was limited for other measurement properties such as reliability, criterion validity, and construct validity. None of the scales reported all psychometric properties outlined by COSMIN. The New Short-Form of the Premenstrual Symptoms Questionnaire and the Japanese version of the Daily Record of Severity of Problems demonstrated sufficient structural validity and internal consistency, although the quality of evidence for other properties was indeterminate.

**Conclusions:**

Although some PROMs demonstrated promising psychometric properties, further validation studies are required for most scales. The development of innovative scales with robust measurement properties is essential for advancing the assessment of PMS/PMDD in Japanese clinical and research settings. Careful consideration of the characteristics of each PROM is necessary when selecting instruments for specific purposes.

**Supplementary Information:**

The online version contains supplementary material available at 10.1186/s41687-025-00910-4.

## Background

Premenstrual syndrome (PMS) is a condition in which various symptoms occur repeatedly 3–10 days prior to each menstrual bleeding cycle [1]. The most common symptoms include breast tenderness; bloating; headache; and mood-related changes such as mood swings, depression, anxiety, anger, and irritability. Premenstrual dysphoric disorder (PMDD), a severe form of PMS, is characterized predominantly by clinically significant emotional and affective symptoms, such as mood swings, depression, anxiety, anger, and irritability, that are not attributable to another psychiatric condition [2, 3]. PMS and PMDD are significant health problems in menstruating women. The American Psychiatric Association initially introduced PMDD as a research criterion in the fourth edition of the Diagnostic and Statistical Manual of Mental Disorders (DSM) in 1994 [4]. Subsequently, PMDD was categorized in the DSM-5 using depressive disorders as part of its diagnostic criteria [5]. PMS/PMDD symptoms fluctuate across cycles and require prospective tracking for an accurate diagnosis. Therefore, current guidelines recommend using a daily symptom chart recorded over at least two menstrual cycles [5–7]. According to a recent meta-analysis, the global prevalence of PMS is high, and approximately half of the women of reproductive age experience PMS [8]. Global PMS/PMDD prevalence estimates vary significantly by study methodology. Western prospective studies report PMS around 20–30% and PMDD prevalence around 3–8% [9], reflecting the predominant research focus on these populations. Comparatively limited data exist for Asia; examples include Japan (5.3% moderate-to-severe PMS / 1.2% PMDD, retrospective) [10] and China (21.1% PMS / 2.1% PMDD, prospective) [11]. Although these Asian PMDD rates appear closer to the lower end of Western estimates, methodological variations (e.g., study design, questionnaires, recall period) and data scarcity prevent definitive conclusions regarding regional disparities [12]. Consistent, prospective research across diverse populations is therefore warranted.

The conditions of PMS/PMDD range from pre-treatment health problems to treatment-resistant serious illnesses, all of which reduce the health-related quality of life. Given the increasing availability of both medical and non-medical healthcare services for PMS/PMDD, as well as the fact that symptoms include psychological components, patient-reported outcomes have become vital for assessing symptom presentation, determining the necessity of therapeutic interventions, and evaluating their effectiveness. Various patient-reported outcome measures (PROMs) have been developed to evaluate PMS/PMDD in Japanese populations based on study objectives; however, neither the quality of evidence for specific PROMs has been systematically evaluated nor has their synthesis been clearly established. Selecting the most suitable PROM requires the consideration of specific characteristics, including interpretability, feasibility, and measurement properties. Consolidating existing evidence on these PROMs is essential for this purpose. Furthermore, summarizing the findings from individual countries is a critical step toward contributing to international evidence and fostering a cross-cultural understanding of PMS/PMDD.

The COnsensus-based Standards for the selection of health Measurement Instruments (COSMIN) provides a comprehensive and widely accepted framework for the evidence-based selection of PROMs tailored to specific constructs and populations [13]. For the PROMs of PMS/PMDD to be useful and robust, they must demonstrate sufficient quality in terms of measurement properties [14]. The COSMIN initiative developed consensus-based criteria and evaluation standards for assessing the quality of PROMs and considered the following important measurement properties: content validity, structural validity, internal consistency, construct validity, reliability, measurement error, cross-cultural validity/measurement invariance, criterion validity, and responsiveness [13, 14]. Evaluating and synthesizing the measurement properties of these instruments play a key role in supporting the evidence-based selection of PROMs.

Therefore, we conducted a systematic review of PROMs used to assess PMS/PMDD in the Japanese population. This study aimed to evaluate these instruments in terms of their purpose, methodological quality, and measurement properties as well as determine whether their reported psychometric properties satisfy the acceptable standards of measurement properties.

## Methods

This systematic review was conducted according to the COSMIN guideline for systematic reviews [13], which provides a framework for evaluating measurement instruments and assessing the risk of bias in PROMs [13, 14]. This study was registered in PROSPERO (registration number: CRD42024552198).

### Data sources and literature search strategy

The MEDLINE (PubMed), CINAHL (www.ebsco.com/), Cochrane Library (www.cochranelibrary.com/), and Ichushi-Web (www.jamas.or.jp/) databases were searched on August 14, 2024.

To ensure comprehensive coverage, the bibliographies of the selected articles were manually examined to identify additional relevant studies and available translations.

The search strategy was developed by an information librarian using a wide range of search terms and measurement properties for the PMS/PMDD assessment tool. The search strategy comprised four key components: (1) measurement properties, including terms related to reliability and validity (e.g., “reliabilities,” “valid,” “validation”); (2) tools for measurement, including terms such as “measure,” “assessment,” “questionnaire,” and “scale”; (3) the constructs of interest, focusing on PMS and PMDD, with terms like “Premenstrual Syndrome,” “Premenstrual Dysphoric Disorder,” and related keywords; and (4) the geographical context, specifically targeting studies conducted in Japan, using the term “Japanese.” No specific search terms were used for the broader construct, as the goal was to capture all validated instruments related to PMS/PMDD. The full search strategy for each database is available in Appendix 1. Studies were eligible for inclusion if they evaluated at least one measurement property of a PROM developed to assess PMS or PMDD in Japanese populations. Included studies focused on women in Japan with menstrual cycles and used self-administered or interview-based instruments. Only full-text publications written in Japanese or English, including peer-reviewed journal articles and academic books, were considered. Studies that used PROMs solely as comparison instruments in the validation of other instruments were excluded. The selection process followed the Preferred Reporting Items for Systematic Reviews and Meta-Analyses 2020 guidelines [15].

### Selection of studies

The databases were systematically examined using pre-specified search terms to generate the initial search results. Duplicate articles were removed prior to the procedure. Initial screening of titles and abstracts was performed by one author (KT). Studies identified by KT as potentially relevant or irrelevant based on the inclusion/exclusion criteria proceeded accordingly. For studies where eligibility was uncertain based on the title and abstract, they were independently assessed by a second author (TO). Disagreements between KT and TO regarding these uncertain studies, or cases requiring further clarification after the independent assessment by TO, were resolved through discussion and consensus involving additional reviewers. Following the title and abstract screening, all potentially eligible studies underwent full-text review by all five authors to determine final inclusion. Inter-rater reliability during the full-text screening stage was assessed using Fleiss’ Kappa (κ), calculated with the statsmodels package (version 0.14.4) in Python (version 3.11.12). Discrepancies were resolved through a consensus meeting.

### Data extraction

The characteristics of the included PROMs (e.g., construct, target population, recall period, and length of instrument), information on the feasibility of the included PROMs, and information on interpretability were extracted. Data extraction was performed by KT and checked by TO, YI, YT, and HS. Data were recorded using templates and output tables available online [13, 14, 16].

Subsequent steps were conducted one measurement property at a time in the following order, as per the COSMIN guideline [13]: structural validity, internal consistency, cross-cultural validity/measurement invariance, reliability, measurement error, criterion validity, hypotheses testing for construct validity, and responsiveness. The most important measurement characteristic of COSMIN is content validity, and guidelines have been developed to independently evaluate the quality of content validity [17]. Therefore, this review focused solely on the measurement properties, excluding content validity.

### Evaluation of the methodological quality of the included studies

The methodological quality of the included studies was assessed by four of the five independent authors (KT, TO, YI, YT, and HS) using the COSMIN Risk of Bias checklist, resulting in “very good,” “adequate,” “doubtful,” or “inadequate” ratings for each study [14]. Because YI contributed to several of the reviewed articles, she abstained from evaluating the literature in which she was closely involved. Discrepancies among the reviewers were resolved through a consensus meeting. For each measurement property in each study, the overall rating was determined based on the “worst score counts” principle—that is, the lowest rating among the relevant items was assigned as the final score for that property.

### Assessment of psychometric properties

The results of each study on a measurement property were rated against the criteria for good measurement properties, resulting in a “sufficient” (+), “insufficient” (–), or “indeterminate” (?) rating [13]. The “indeterminate” (?) rating was applied when a result could not be properly evaluated, typically due to either inadequate reporting within the study (e.g., missing statistical details) or non-fulfillment of essential methodological requirements (such as assessing internal consistency without adequate evidence of structural validity). Studies examining hypothesis testing for construct validity were evaluated with careful consideration of heterogeneity among the scales related to PMS/PMDD. Given this diversity, the review team refrained from pre-establishing hypotheses regarding these relationships. Instead, comparisons made in studies between PMS/PMDD-related scales and other relevant scales were adopted as hypothesis testing for construct validity. The gold standard for assessing criterion validity in this review was defined as “a physician’s judgment based on two or more cycles of prospective symptom documentation” [5, 6, 18]. Reliability was evaluated separately for the luteal and follicular phases, as the results may vary between these phases. Four of the five independent authors—KT, TO, YT, and HS—performed each quality evaluation step. Disagreements among individual authors’ judgments were discussed with all authors to reach a consensus.

### Evidence synthesis and certainty assessment

If two or more studies existed for a single PROM, evidence from individual studies on the same PROM or subscale was summarized per measurement property. Individual ratings for each measurement property were qualitatively synthesized using priori rules based on COSMIN recommendations. Based on these rules, each PROM received an overall (synthesized) rating of “sufficient’’ (+), “insufficient” (–), or “inconsistent” (±) for each measurement property. The quality of evidence per measurement property per PROM was graded using a modified Grading of Recommendations Assessment, Development and Evaluation (GRADE) approach, resulting in “high,” “moderate,” “‘low,” and “very low” grades [13]. The modified GRADE approach was used to downgrade the evidence when there were concerns about the quality of the evidence based on four factors: risk of bias, imprecision, inconsistency, and indirectness. The quality of evidence was not graded for studies in which the overall rating was indeterminate (?) or inconsistent (±). If only one study exists for a single PROM, it is impossible to evaluate whether the quality of evidence is consistent across studies. Therefore, the modified GRADE approach was not applied (no level of evidence: rating is indeterminate). Both KT and TO independently completed this step, and discrepancies were resolved through discussions among all authors.

## Results

### Study selection

**Fig. 1:**
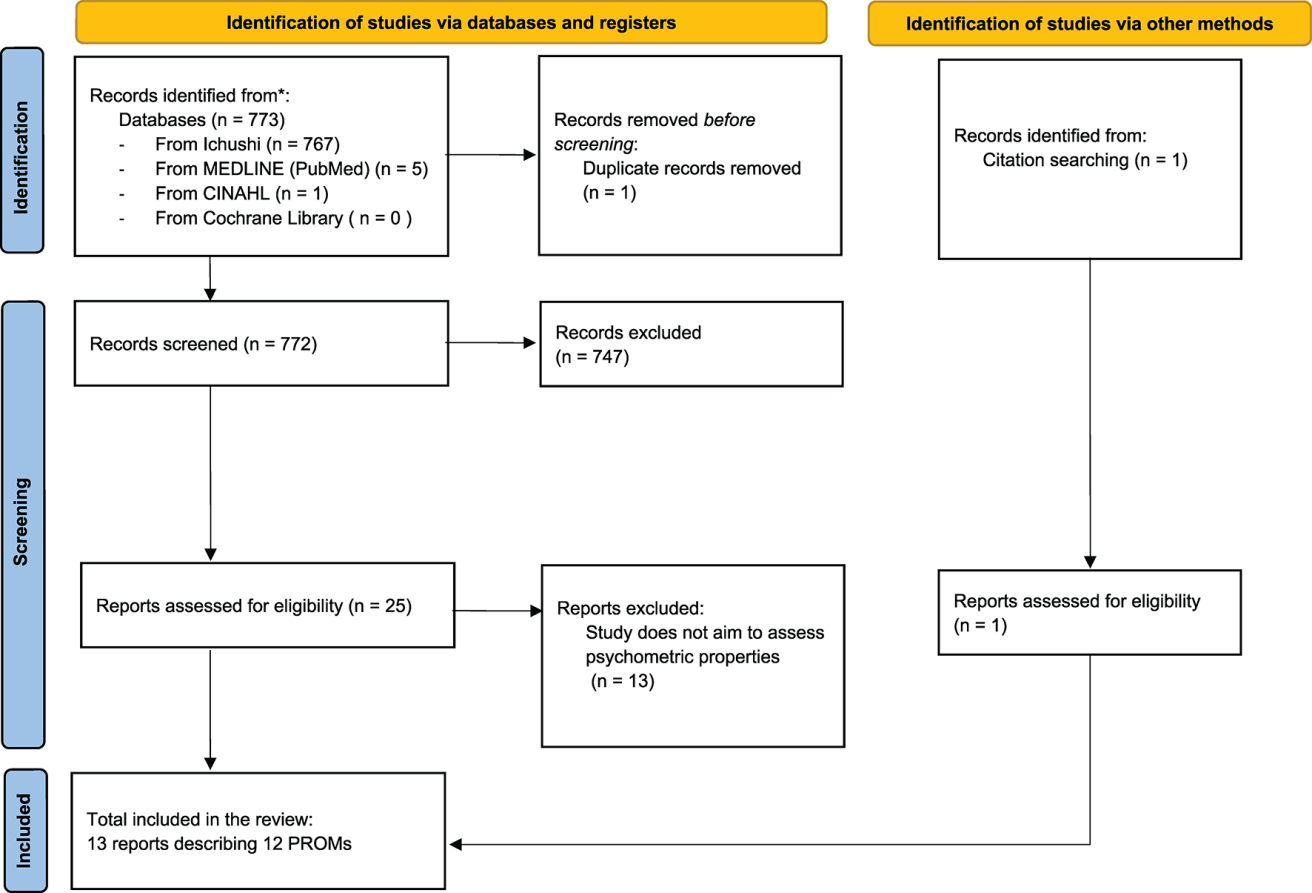
PRISMA Flow diagram. The full search strategy is provided online in supplemental information 1. PRISMA, Preferred Reporting Items for Systematic Reviews and Meta-Analyses; PROM, patient-reported outcome measure

The database search and reference check resulted in 772 unique abstracts, of which 26 full-text articles were assessed for eligibility. Inter-rater agreement during the full-text screening phase was retrospectively assessed after the review process, with a Fleiss’ Kappa coefficient of 0.88, indicating almost perfect agreement among the five reviewers. Ultimately, 13 articles describing 12 versions of 11 unique PROMs were included in this review. A flowchart is presented in Fig. 1. The characteristics of included PROMs are listed in Table 1. The scales were categorized into two groups based on their recording method and recall period: nine (69%) were classified as recall-based, and four (31%) were classified as daily recording scales. Recall-based scales retrospectively assess premenstrual symptoms at a single time point, whereas daily recording scales involve recording symptoms daily and using data specifically from the luteal phase.

**Table 1:** Characteristics of the included proms

PROM (reference to the first article)	Constructs	Target population	Mode of administration (e.g., self-report, interview-based, or parent/proxy report)		Recall period	Length of the instrument	Original language	Available translations
PMDD scale^13^	Screening tool for patients with suspected PMDD	-		Self-report	1 year	2 subscales, 17 items: subscales I (12 items) and II (5 items)	Japanese	-
PSQ^14^	Screening tool for PMS	-	Self-report		3 months	2 subscales, 14 items: Symptoms (11) and functional impairment of social and life activities (3)	Japanese	-
PSQ-S^15^	Simple assessment of PMS	-	Self-report		3 months	9 items	Japanese	-
PMS-I^16^	Measuring psychosocial aspects of PMS impact	-	Self-report		-	2 subscales, 13 items: Social (4) and psychological (9) factors	Dutch	-
PSMS^17^	Identification and quantification of psychological distress in women throughout the menstrual cycle due to menstrual symptoms	-	Self-report		-	2 subscales, 20 items: Sense of loss of control (12) and disadvantage (8)	Japanese	-
PMS-8^18^	Measuring the burden of PMS	-	Self-report		-	Two subscales, eight items: Physical (4) and psychological (4) factors	Japanese	-
Self-management scale of PMS during childrearing periods^19^	Measuring the self-management capacity of patients with PMS	Mothers currently raising children		Self-report	-	5 subscales, 48 items: Premenstrual emotional instability (10), positive emotional changes after the onset of menstruation (8), “Mothers” perspective on supports provided by their husbands (partners) before/after the onset of menstruation (6), premenstrual lowered energy (10), and premenstrual physical unpleasant conditions (4)	Japanese	-
37 items of the Premenstrual State Scale^20^	Measuring a wide range of PMS symptoms	Young women	Self-report		-	4 subscales, 37 items: Mental complaints 1 (10); mental complaints 2 (10); physical complaints 1 (10); and physical complaints 2 (7)	Japanese	-
MDQ^21^	Measurement of discomfort associated with the menstrual cycle	-	Self-report		Current	6 subscales, 35 items: Pain (6), concentration (8), behavioural change (5), autonomic reaction (4), water retention (4), and negative effect (8)	Japanese	-
DRSP-J^22,23^	Diagnosis and assessment of PMS and PMDD	-	Self-report		Current	2 subscales, 24 items: Symptoms (21) and dysfunction in daily life (3)	English	Dutch, Portuguese, and Chinese
J-DRSP^24, 25^	Diagnosis and assessment of PMS and PMDD	-	Self-report		Current	2 subscales, 24 items: Symptoms (21) and dysfunction in daily life (3)	English	Dutch, Portuguese, and Chinese
J-DRSP (SF) ^25^	Simple assessment of PMS and PMDD	-	Self-report		Current	2 subscales, 8 items: Physical (4) and psychological (4) factors	English and Japanese	-

PROM, patient-reported outcome measure; PMS, Premenstrual syndrome; PMDD, premenstrual dysphoric disorder; PSQ, Psychometric Testing of the Premenstrual Symptoms Questionnaire; PSQ-S, Psychometric Testing of a New Short-Form of the Premenstrual Symptoms Questionnaire; PMS-I, PMS-Impact Questionnaire; PSMS, psychological sufferings in menstruation-related symptoms; PMS-8, Premenstrual Syndrome Short Scale 8 items; MDQ, Menstrual Distress Questionnaire; DRSP-J/J-DRSP, Japanese version of the Daily Record of Severity of Problems; J-DRSP (SF), Short-Form version J-DRSP

The recall-based scales included the PMDD scale [19], Premenstrual Symptoms Questionnaire (PSQ) [20], New Short-Form of the Premenstrual Symptoms Questionnaire (PSQ-S) [21], Japanese version of PMS-Impact [22], the psychological sufferings in menstruation-related symptoms scale [23], Premenstrual Syndrome Short Scale 8 items [24], Self-Management Scale of Premenstrual Syndrome during Childrearing Periods [25], 37 items of the Premenstrual State Scales [26], and the modified Japanese version of Menstrual Distress Questionnaire (MDQ) [27]. The daily recording scales included the Japanese Version of the Daily Record of Severity of Problems (DRSP-J) [28, 29], the Japanese version of the Daily Record of Severity of Problems (J-DRSP) [30, 31], and the short form of J-DRSP (J-DRSP (SF)) [31]. The DRSP was translated into two versions from the original version of DRSP [32]. All these PROMs are self-reported measures.

Appendix 2 provides the characteristics of study populations involved in the PROM design. Table 2 provides information on the feasibility and interpretability. Notably, DRSP-J required permission from its developer for use [33].

**Table 2:** Information on feasibility and interpretability of proms

PROM	Response options including subscale	Completion time	Required mental and physical ability levels of the patients	Ease of score calculation	Item accessibility in the paper	Distribution of scores in the study population	Percentages of missing items and total scores	Floor and ceiling effects	Scores and changes in scores available for relevant subgroups	Minimal important change or difference	Information on response shift
PMDD scale^13^	None (1 point), a little (2 points), yes (3 points), and very strongly (4 points)	-	-	(1) At least one symptom of “very strongly” exists among symptoms 1–4 in the PMDD Rating Scale I. (2) In addition to (1), at least four symptoms of “yes” or “very strongly” exist among symptoms 1–12 in the PMDD Rating Scale I. (3) At least one symptom of “very strongly” exists among the symptoms of items 1–5 in the PMDD Rating Scale II. The patients who fulfilled the above three conditions are graded as having suspected PMDD.	Accessible	-		-		-	
PSQ^14^	1, not at all; 2, mild; 3, moderate; and 4, Severe.	-	-	The total score is calculated as the sum of 14 items (ranging from 14 to 56).	Inaccessible	-	-	-		-	
PSQ-S^15^	1, not at all; 2, mild; 3, moderate; and 4, severe.	-	-	-	Inaccessible	Total score ranges from 9 to 36.	-	-		-	
PMS-I^16^	1, not at all true; 2, not very true; 3, somewhat true; 4, truly true; and 5, very true.	-	-	-	Only scale items are accessible	Mean, 2.66 and SD, 0.92	-	-		-	
PSMS^17^	1, not at all true; 2, not very true; 3, somewhat true; 4, truly true; and 5, very true.	-	-	-	Only scale items are accessible	-	-	-		-	
PMS-8^18^	1, not at all; 2, very little; 3, mild; 4, moderate; 5, strong; and 6, very strong.	-	-	The total scores are derived by adding the scores of every item (minimum = 1; maximum = 6), resulting in minimum and maximum total scores of 8 and 48, respectively.	Accessible	Mean, 33.4 and SD, 6.8; range, (possible: 8–48) 14–48		-		-	
Self-management scale of PMS during childrearing periods^19^	1, strongly disagree; 2, disagree. 3, neither agree nor disagree; 4, agree; and 5, strongly agree.	Approximately 5 min	-	-	Only scale items are accessible	Mean, 133.3 and SD, 27.7; range, 48–223		-		-	
37 items of the premenstrual state scales^20^	None, once, or two times a year; every 2 or 3 months; or monthly	-	-	-	Only scale items are accessible	-	-	-		-	
MDQ^21^	1, no symptoms; 2, slightly present; 3, mildly present; 4, moderately present; 5, strongly present; and 6, severely disturbed.	-	-	-	Only scale items are accessible	-		-		-	
DRSP-J^22,23^	1 (not at all) to 6 (extreme)	-	-	Total score was calculated as the sum of the 21 premenstrual symptoms, and the total score ranges from 21 to 126.	Fully accessible [26]		-	-		-	
J-DRSP^24,25^	1 (not at all) to 6 (extreme)	The median time in the pilot study was 2 min 5 s.	-		Fully accessible	The median total score of the 24 items in J-DRSP is 34 (range, 24–85).		-		-	
J-DRSP (SF)^25^	1 (not at all) to 6 (extreme)				Only scale items are accessible	-	-	-		-	

PROM, patient-reported outcome measure; PMS, premenstrual syndrome; PMDD, premenstrual dysphoric disorder; SD, standard deviation; PSQ, Psychometric Testing of the Premenstrual Symptoms Questionnaire; PSQ-S, Psychometric Testing of a New Short Form of the Premenstrual Symptoms Questionnaire; PMS-I, PMS-Impact Questionnaire; SD, standard deviation; PSMS, psychological sufferings in menstruation-related symptoms; PMS-8, Premenstrual Syndrome Short Scale 8 items; MDQ, Menstrual Distress Questionnaire; DRSP-J/J-DRSP, Japanese version of the Daily Record of Severity of Problems; J-DRSP (SF), Short-Form version of the J-DRSP

### Measurement properties

Table 3 summarizes the results of the included studies on the measurement properties of PROMs. The methodological quality and the results of these studies are also presented. Appendix 3 provides an overview of the findings and the quality of the evidence. The results for each characteristic are described as follows:

**Table 3:** Summary of the results of the included studies on measurement properties per PROM

**PROM subscale**	Structural validity			Internal consistency			Cross-cultural validity/measurement invariance			Reliability			Measurement error			Criterion validity			Hypothesis testing a: comparison with other instruments; b: comparison between subgroups			Responsiveness		
	n	methqual**	Result (rating) *	n	methqual**	Result (rating) *	n	methqual	Result (rating) *	n	methqual**	Result (rating) *	n	methqual**	Result (rating) *	n	methqual**	Result (rating) *	n	methqual**	Result (rating) *	n	methqual**	Result (rating) *
PMDD scale^13^	303	Very good	(-)	303	Very good	(+)													66	a: doubtful	(?)			
PSQ^14^	879	Very good	(+)	879	Inadequate	(+)													879	a: doubtful; b: doubtful	(?)			
PSQ-S^15^	922	Very good	(+)	922	Very good	(+)										922	Inadequate	(+)	922	a: doubtful	(?)			
PMS-I^16^	254	Adequate	(?)	254	Very good	(+)													254	b: very good	(+)			
PSMS^17^	318	Very good	(-)	318	Very good	(+)													318	a: very good	(+)			
PMS-8^18^	415	Very good	(-)	415	Very good	(-)													415	a: doubtful	(?)			
Self-management scale of PMS during childrearing periods^19^	797	Adequate	(?)	797	Very good	(+)													797	a: very good; b: adequate	(?)			
37 items of the premenstrual state scales^20^	477	Doubtful	(?)	477	Very good	(+)																		
MDQ^21^	34	Inadequate	(?)	34	Very good	(-)													34	b: inadequate	(?)			
DRSP-J^22^	324	Very good	(+)	324	Very good	(+)				324	Inadequate	(+)							324	a: doubtful	(?)			
DRSP-J^23^	113	Adequate	(?)	113	Very good	(+)				113	Adequate	(-) (in the mid-follicular phase); (+) (in the late luteal phase)				113	Very good	(+)	113	a: doubtful	(?)			
J-DRSP^25^	228	Very good	(-)	228	Very good	(+)				228	Adequate	(+) (in follicular phase); (-) (in luteal phase).												
J-DRSP^24^				107	Very good	(-)													107	a: doubtful	(?)			
J-DRSP (SF)^25^	228	Very good	(+)	228	Very good	(+)				228	Adequate	(+) (in the follicular phase) (-); (in the luteal phase).				228	Very good	(+)						

PROM, patient-reported outcome measure; PMS, premenstrual syndrome; PMDD, premenstrual dysphoric disorder; PSQ, Psychometric Testing of the Premenstrual Symptoms Questionnaire; PSQ-S, Psychometric Testing of a New Short Form of the Premenstrual Symptoms Questionnaire; PMS-I, PMS-Impact Questionnaire; PSMS, psychological sufferings in menstruation-related symptoms; PMS-8, Premenstrual Syndrome Short Scale 8 items; MDQ, Menstrual Distress Questionnaire; DRSP-J/J-DRSP, Japanese version of the Daily Record of Severity of Problems; J-DRSP (SF), Short-Form version of the J-DRSP

* The result of each study on a measurement property of a PROM was rated against the updated criteria for good measurement properties: (+), sufficient; (−), insufficient; (±), inconsistent; (?), indeterminant

** The result of each study on the methodological quality assessment: Very good, Adequate, Doubtful, Inadequate

### Structural validity

All included PROMs were evaluated for structural validity. The PSQ, PSQ-S, DRSP-J, and J-DRSP (SF) were found to have sufficient structural validity. However, the structural validity of other PROMs did not satisfy the COSMIN criteria for good measurement properties.

### Internal consistency

Internal consistency ratings were confirmed for all PROMs. Sufficient internal consistency was found for the PMDD scale, PSQ-S, PMS-Impact, Self-Management Scale of Premenstrual Syndrome during Childrearing Periods, DRSP-J, J-DRSP, and J-DRSP (SF).

### Reliability

The reliability was evaluated using three daily recording scales: DRSP-J, J-DRSP, and J-DRSP (SF). No scale demonstrated sufficient reliability in either the follicular or luteal phase. No scale assessed test-retest reliability over more than two cycles.

### Criterion validity

Three PROMs were evaluated for criterion validity: DRSP-J, J-DRSP, and J-DRSP (SF). The two short versions were compared with the long version of the scale. In one study, only the DRSP-J was compared to the gold standard, and its criterion validity was evaluated [29]. Furthermore, the DRSP-J was categorized into three groups, and the agreement rate with the three categories of clinical judgment was evaluated. Although this method does not directly correspond to either continuous or dichotomous scores in the bias risk evaluation, it was deemed methodologically appropriate to calculate the association between the DRSP categories and clinical judgment using the Kappa coefficient.

### Hypothesis testing for construct validity

Nine studies identified associations between PROMs that assessed PMS/PMDD and other instruments [20–25, 28–30]. These studies tested the hypotheses for construct validity. However, limited evidence regarding the psychometric properties of comparator scales precluded an adequate assessment of their methodological quality.

### Remaining measurement properties

Cross-cultural validity/measurement invariance, measurement error, and responsiveness were not evaluated in any of the studies.

## Discussion

This study systematically evaluated the measurement properties of existing PROMs for assessing PMS/PMDD in Japan. We followed the COSMIN guideline for systematic reviews to ensure a high-quality systematic review with reliable results [13, 34]. In this review, 12 versions of 11 unique PROMs were evaluated. Most of the scales exhibited adequate structural validity and internal consistency. However, there was limited evidence regarding its reliability, criterion validity, and construct validity. The PSQ-S and J-DRSP (SF) demonstrated sufficient structural validity and internal consistency, although the evidence for other properties was inconclusive.

The 12 scales evaluated in this review were broadly categorized into recall-based and daily recording scales based on their recall periods. Daily recording, the diagnostic gold standard for confirming cyclical symptoms, minimizes recall bias and captures symptom timing accurately; however, it imposes a significant user burden and faces compliance challenges. In contrast, recall-based scales offer greater convenience and lower burden by assessing symptoms retrospectively at one point, making them suitable for screening or clinical studies. However, this convenience comes at the cost of potential recall bias, influenced by memory and current mood, which can compromise the accurate assessment of symptom patterns compared to daily diaries.

However, the consistency between daily recordings and recall-based scales has been reported to be suboptimal [35]. This discrepancy may be attributed not only to recall bias, particularly in retrospective assessments, but also to inherent variability in symptoms across menstrual cycles, which is a defining characteristic of PMS/PMDD. Importantly, PMS/PMDD assessment is more accurately captured through daily recording scales, making them the preferred choice for intervention studies aimed at evaluating treatment effects. Conversely, recall-based scales are more practical for use in cross-sectional studies. However, these scales tend to reflect the perceived burden of PMS/PMDD symptoms rather than their objective severity, focusing more on the individual’s subjective awareness of their condition.

None of the scales reported all the psychometric properties outlined by the COSMIN methodology in this study. Structural validity and internal consistency were relatively well evaluated, with evidence for internal consistency often demonstrating high quality. However, standardized instruments to assess construct validity are lacking. This poses significant challenges for the hypothesis testing of construct validity in Japanese PMS/PMDD scales, making it inherently difficult to establish unified hypotheses across studies. Future research should categorize studies using specific and appropriate comparator scales to facilitate robust validity assessments.

Most of the reviewed scales lacked appropriate methodologies to assess critical measurement properties such as reliability and measurement error. Although some of the scales are acceptable in terms of reliability, they were not evaluated across different menstrual phases. Recall-based scales have not been assessed for reliability and failed to capture temporal changes.

Furthermore, criterion validity has rarely been evaluated against the gold standard, which involves physicians’ judgment based on prospective symptom documentation over two or more cycles. In addition to these challenges, psychometric properties often remain limited in scope, with critical aspects such as cross-cultural validity/measurement invariance and responsiveness being insufficiently addressed. The lack of cross-cultural validity and measurement invariance raises concerns about the generalizability of these scales to Japanese populations or subgroups, limiting their applicability in diverse cultural contexts. Similarly, a lack of responsiveness evaluation prevents confirmation of these scales’ ability to detect meaningful change, thereby limiting their confident use for tracking outcomes in clinical and research settings.

This review highlights the gaps in the evidence for the psychometric evaluation of PMS/PMDD scales. Notably, most scales were examined in only a single study, which limits the robustness and generalizability of the evidence supporting these instruments.　Widely utilized scales such as the Japanese version of the MDQ [36] and the premenstrual symptom screening tool [37] were excluded from this review as they did not meet the inclusion criteria requiring published psychometric evaluations. Specifically, although a Japanese version of the MDQ has been published [38], its reliability and validity were not assessed, and no publication evaluating the psychometric properties of a Japanese version of the PSST could be identified. This lack of robust evidence highlights the need not only for validating existing scales but also for exploring novel approaches to address the limitations of current scales. Novel scales with robust psychometric properties are expected to be developed to enhance their utility in clinical and research contexts in Japan. Since PMS/PMDD forms a spectrum where even subthreshold symptoms are often impairing [39], assessment should not be limited to diagnostic thresholds. Thus, novel scales are needed to evaluate subthreshold PMS and provide immediate, continuous assessment, thereby enabling a more comprehensive clinical evaluation.

Addressing these challenges will enhance the accuracy of diagnostic and screening practices, contribute to more effective interventions, and improve the outcomes of individuals affected by these conditions.

### Limitations

This study has some limitations. First, the number of included studies was small, with only one study available for review on most scales at any given time point. Despite this limitation, this review synthesized the best available evidence in an understudied field, providing a valuable foundation for future research and identifying key areas for further investigation. Second, the literature search for Japanese studies was conducted using only a few databases, which may have restricted the scope of this review. Nevertheless, the selected databases are widely recognized and comprehensive for Japanese publications, ensuring that the included studies are both relevant and of high quality. Third, key stages of the review process, including the initial title/abstract screening and data extraction, were primarily conducted by a single reviewer, potentially introducing bias or limiting the robustness of the evaluation process. Regarding the title/abstract screening, systematic dual review of a subset to comprehensively assess inter-rater reliability was not performed. However, to mitigate potential bias at this stage, studies deemed uncertain by the primary reviewer underwent independent assessment by a second author, with unresolved cases discussed for consensus among additional reviewers. For data extraction, potential bias was addressed through checks by other reviewers and adherence to a clearly defined and systematic methodology for data extraction and analysis. Furthermore, detailed documentation of the entire review process ensures transparency and facilitates reproducibility. Finally, because COSMIN has developed guidelines to independently assess the quality of content validity [17], this study did not assess the content validity of the included PROMs. Future research should prioritize the evaluation of the content validity of these instruments by focusing on their relevance, comprehensiveness, and comprehensibility in PROMs.

## Conclusion

In conclusion, we identified 12 versions of 11 unique PROMs for assessing PMS/PMDD in Japanese. Although the quality of evidence for many other measurement properties was low, the PSQ-S and J-DRSP (SF) demonstrated sufficient structural validity and internal consistency. However, further validation of existing scales and the development of innovative assessment scales with robust psychometric properties are essential to advance their application for assessing PMS/PMDD in both clinical and research settings in Japan. In addition, it is crucial to understand the characteristics of each existing PROM and select the most appropriate PROM according to its intended use.

## Electronic supplementary material

Below is the link to the electronic supplementary material.

Supplementary Material 1

Supplementary Material 2

Supplementary Material 3

## Data Availability

All data generated or analyzed during this study are included in this published article and its supplementary information files.

## Abbreviations

PROM: Patient-reported outcome measure
PMS: Premenstrual synrdrome
PMDD: Premenstrual dysphoric disorder
COSMIN: COnsensus-based Standards for the selection of health Measurement Instruments
MDQ: Modified Japanese version of Menstrual Distress Questionnaire
PSQ: Premenstrual Symptoms Questionnaire
PSQ-S: New Short-Form of the PSQ
J-DRSP/DRSP-J: Japanese Version of the Daily Record of Severity of Problems
J-DRSP (SF): Short form of J-DRSP
