# Supplementary material for: Psychometric properties of patient-reported outcome measures to assess premenstrual syndrome/premenstrual dysphoric disorder in japanese: a systematic review using the COSMIN methodology
Source: J Patient Rep Outcomes. 2025 Jun 20;9:71. doi: 10.1186/s41687-025-00910-4 (PMC12181441; doi:10.1186/s41687-025-00910-4)
Supplement: Supplementary file 1 — Supplementary Material 1 [file 41687_2025_910_MOESM1_ESM.docx]

PubMed

#1

(("reliabilities"[All Fields] OR "reliability"[All Fields] OR "reliable"[All Fields] OR "reliablity"[All Fields] OR "reliably"[All Fields] OR "valid"[All Fields] OR "validate"[All Fields] OR "validated"[All Fields] OR "validates"[All Fields] OR "validating"[All Fields] OR "validation"[All Fields] OR "validational"[All Fields] OR "validations"[All Fields] OR "validator"[All Fields] OR "validators"[All Fields] OR "validities"[All Fields] OR "validity"[All Fields])

#2

("measure*"[All Fields] OR "assessment*"[All Fields] OR "questionnaire*"[All Fields] OR "scale*"[All Fields] OR "index*"[All Fields] OR "instrument*"[All Fields] OR "tool*"[All Fields]))

#3

("Premenstrual dysphoric disorder"[mesh] OR "Premenstrual dysphoric disorder"[tiab] OR "Disorder, Premenstrual Dysphoric"[tiab] OR "Dysphoric Disorder, Premenstrual"[tiab] OR "Premenstrual Dysphoric Syndrome"[tiab] OR "Syndrome, Premenstrual Dysphoric"[tiab] OR PMDD[tiab] OR "Premenstrual Syndrome"[mesh] OR "Premenstrual Syndromes"[tiab] OR "Syndrome, Premenstrual"[tiab] OR "Premenstrual Tension"[tiab] OR "Premenstrual Tensions"[tiab] OR "Tension, Premenstrual"[tiab] OR "late luteal phase dysphoric disorder"[tiab] OR "LLPDD"[tiab])

#4

(“Japanese” [All Fields])

CINAHL

#1

"reliabilities" OR "reliability" OR "reliable" OR "reliablity" OR "reliably" OR "valid" OR "validate" OR "validated" OR "validates" OR "validating" OR "validation" OR "validational" OR "validations" OR "validator" OR "validators" OR "validities" OR "validity"

686,159件

#2

"measure*" OR "assessment*" OR "questionnaire*" OR "scale*" OR "index*" OR "instrument*" OR "tool*"

2,456,686件

#3

MH "Premenstrual dysphoric disorder" OR "Premenstrual dysphoric disorder" OR "Disorder, Premenstrual Dysphoric" OR "Dysphoric Disorder, Premenstrual" OR "Premenstrual Dysphoric Syndrome" OR "Syndrome, Premenstrual Dysphoric" OR “PMDD” OR MH "Premenstrual Syndrome+" OR "Premenstrual Syndromes" OR "Syndrome, Premenstrual" OR "Premenstrual Tension" OR "Premenstrual Tensions" OR "Tension, Premenstrual" OR "late luteal phase dysphoric disorder" OR "LLPDD"

2,074件

#4

Japanese

Cochrane Library

#1

reliabilities OR reliability OR reliable OR reliablity OR reliably OR valid OR validate OR validated OR validates OR validating OR validation OR validational OR validations OR validator OR validators OR validities OR validity

#2

measure* OR assessment* OR questionnaire* OR scale* OR index* OR instrument* OR tool*

#3

("Premenstrual dysphoric disorder" OR "Disorder, Premenstrual Dysphoric" OR "Dysphoric Disorder, Premenstrual" OR "Premenstrual Dysphoric Syndrome" OR "Syndrome, Premenstrual Dysphoric" OR “PMDD” OR "Premenstrual Syndromes" OR "Syndrome, Premenstrual" OR "Premenstrual Tension" OR "Premenstrual Tensions" OR "Tension, Premenstrual" OR "late luteal phase dysphoric disorder" OR "LLPDD"):ti,ab OR [mh “Premenstrual Dysphoric Disorder”] OR [mh “Premenstrual Syndrome”]

#4

Japanese

Ichushi web

(((((月経前症候群/TH or 月経前症候群/AL)) or ((月経前不快気分障害/TH or 月経前不快気分障害/AL)) or ((月経困難症/TH or 月経困難症/AL)) or ((月経異常/TH or 月経異常/AL)) or (LLPDD/AL) or (late/AL and luteal/AL and phase/AL and dysphoric/AL and (疾患/TH or disorder/AL)) or (黄体後期不機嫌性障害/AL)) and (((評価基準/TH or 評価基準/AL)) or ((質問紙法/TH or 質問紙法/AL)) or ((調査と質問紙法/TH or 調査と質問紙法/AL)) or ((自己報告式質問調査/TH or 自己報告式質問調査/AL)) or (尺度/AL))) and (DT=1960:2024 and PT=原著論文))
